# What is the frequency of floor of the mouth lesions? A descritive study of 4,016 cases

**DOI:** 10.4317/medoral.24537

**Published:** 2021-10-27

**Authors:** Anderson Mauricio Paiva e Costa, Flávia Sirotheau Corrêa Pontes, Lucas Lacerda de Souza, Márcio Ajudarte Lopes, Alan Roger Santos-Silva, Pablo Agustin Vargas, Bruno Augusto Benevenuto de Andrade, Kelly Tambasco Bezerra, Mário José Romañach, Ricardo Santiago Gomez, Rafael Ferreira e Costa, Décio dos Santos Pinto Júnior, Danyel Elias da Cruz Perez, Jurema Freire Lisboa de Castro

**Affiliations:** 1 Service of Oral Pathology, João de Barros Barreto University Hospital, Federal University of Pará, Belém/Brazil; 2 Oral Diagnosis Department (Pathology and Semiology Areas), Piracicaba Dental School, University of Campinas, Piracicaba/Brazil; 3 Department of Oral Diagnosis and Pathology, School of Dentistry, Federal University of Rio de Janeiro, Rio de Janeiro/Brazil; 4 Department of Oral Surgery and Pathology, School of Dentistry, Federal University of Minas Gerais, Belo Horizonte/Brazil; 5 Oral Pathology Department, Dental School, University of São Paulo, São Paulo/Brazil; 6 Oral Pathology Department, Dental School, Federal University of Pernambuco, Recife/Brazil; 7 Department of Pathology and Legal Medicine, Federal University of Amazonas, Manaus/Brazil; 8 Health Care Department, Universidad Autónoma Metropolitana Xochimilco, Mexico City/Mexico; 9 Department of Oral Pathology, Dental School, National University of Córdoba, Córdoba/Argentina; 10 The School of Clinical Dentistry, Sheffield/UK

## Abstract

**Background:**

The aim of this study was to investigate the frequency of oral lesions in the floor of the mouth from representative oral pathology centres in Latin America.

**Material and Methods:**

This study was conducted on biopsies obtained from January of 1978 to December of 2018 at nine Latin America oral and maxillofacial pathology centres. Gender, age and histopathological diagnosis were evaluated. Data were analysed using descriptive methods. Chi-square test was used for pairwise comparisons.

**Results:**

From 114,893 samples, 4,016 lesions (3.49%) occurred in the floor of the mouth. Brazil showed 3,777 cases (94%), Mexico 182 cases (4.5%) and Argentina 57 cases (1.4%). Benign lesions represented 65.1% (2,617 cases), followed by 34.9% (1,404 cases) of malignant disorders. Lesions of epithelial origin were more frequent (1,964 cases; 48.9%), followed by salivary glands (1,245 cases; 31%) and soft tissue lesions (475 cases; 11.7%). The most common histological subtypes were oral squamous cell carcinoma (1,347 cases; 33.5%), ranula (724 cases; 18%), oral leukoplakia (476 cases; 11.8%) and inflammatory fibrous hyperplasia (239 cases; 5.9%). The lesion affected males in 2,129 cases and females in 1,897 cases.

**Conclusions:**

In the current study, lesions in the floor of the mouth represented 3.49% of biopsies submitted to oral pathology services and oral squamous cell carcinoma, ranula and leukoplakia were the most common lesions.

** Key words:**Epidemiology, floor of the mouth, benign, malignant.

## Introduction

Floor of the mouth (FOM) is a horizontally aligned U-shaped space situated in the part of the oral cavity that lies beneath the tongue (1). It is commonly associated with a wide variety of local and systemic pathologies and, therefore any FOM lesion is difficult to be identified and usually prompt the patients to ask for medical advice when lesions present any clinical sign or symptom (2). This anatomical site is very susceptible to numerical extrinsic factors, including food, tabacco or alcool associated with tabacco, leading to the development of potentially malignant lesions or/and malignant lesions (2,3).

Epidemiological studies have shown that FOM lesions, including tumours, are commonly found in the clinical practice (3-5). These information are of major importance for constructing the differential diagnosis that will precept diagnostic work-up and management (4). In the recent years, a few studies have explored the prevalence of FOM lesions, however, they showed very heterogeneous results worldwide (3-5).

Regarding the group of lesions, salivary gland tumors only rarely are diagnosed in the FOM, but when it occurs they are usually recognized as malignant neoplasms, demonstrating the importance of determining the distribution of lesions that more frequently involve this region (2). Moreover, oral leukoplakia and oral squamous cell carcinoma are two other important conditions that commonly affect the FOM, while lymphomas are only occasionally identified (4). On the other hand, benign and/or reactive diseases can also be frequently diagnosed and include ranula and epidermoid/dermoid cysts (1,2). Therefore, it becomes very important for clinicians and diagnosticians to understand the distribution of lesions that more commonly affect the FOM in order to better determine the most appropriate diagnostic and/or therapeutic approach. Thus, the aim of this study is to evaluate the clinical and histopathological observation of the lesions affecting the FOM from nine representative Latin America oral and maxillofacial pathology centres. In addition, we will investigate whether the age and gender of the patient raise the diagnostic hypothesis of the lesion.

## Material and Methods

- Study design and ethical approval

This study was performed in the files from nine independent oral and maxillofacial diagnostic centres from Latin America. The biopsies of the centres were retrieved up to a period of 40-years, from January of 1978 to December of 2018. A total of 114,893 samples were analysed. The diagnosis centres were from Brazil (Service of Oral Pathology, João de Barros Barreto University Hospital, Federal University of Pará, Belém/Brazil; Oral Diagnosis Department (Pathology and Semiology), Piracicaba Dental School, University of Campinas, Piracicaba/Brazil; Oral Pathology, Dental School, Federal University of Rio de Janeiro, Rio de Janeiro/Brazil; Department of Oral Surgery and Pathology, School of Dentistry, Federal University of Minas Gerais, Belo Horizonte/Brazil; Oral Pathology Department, Dental School, University of São Paulo, São Paulo/Brazil; Oral Pathology Department, Dental School, Federal University of Pernambuco, Recife/Brazil; Department of Pathology and Legal Medicine, Federal University of Amazonas, Manaus/Brazil), Mexico (Oral Pathology Private Clinics, Coyoacán/Mexico) and Argentina (Department of Estomatology, Dental School, National University of Córdoba, Córdoba/Argentina). The expert oral pathologists of each centre evaluated the samples. The ethical committee of the João de Barros Barreto University Hospital approved this work under approval number 3.381.233. The patient’s identity remained anonymous according to the Declaration of Helsinki.

- Samples

Lesions in the FOM were retrieved, and each of the analysed centres recovered data regarding age, sex and final histopathological diagnosis. All lesions were reclassified according to the World Health Organisation Classification of Head and Neck Tumours Update published in 2017 (6,7). The exclusion criteria included samples that did not present lesion in the FOM, as well as those whose final diagnosis was not possible to be assessed or the diagnosis did not follow the diagnostic criteria of classification established on the referenced textbooks.

- Data analysis

The collected data were tabulated in Microsoft Excel® for epidemiologic analysis. Descriptive and quantitative data analysis was performed and association between categorical variables, e.g. gender, age in decades and final diagnosis were evaluated by Chi-square and Fisher’s exact tests for pairwise comparisons. A P-value <0.05 was considered statistical significant. Statistical analysis was performed with the Statistical Package for Social Sciences (SPSS) software, version 22.0 (SPSS Inc., Chicago, IL, USA).

## Results

From 114,893 samples, 4,016 (3.49%) were in the FOM. Brazil showed 3,777 cases (94%), Mexico 182 cases (4.5%) and Argentina 57 cases (1.4%). Table 1 and Table 2 show the distribution of lesions according to gender and age, respectively. Benign disorders represented 65% (2,613 cases), followed by 35% (1,403 cases) of malignancies.

**Table 1:**
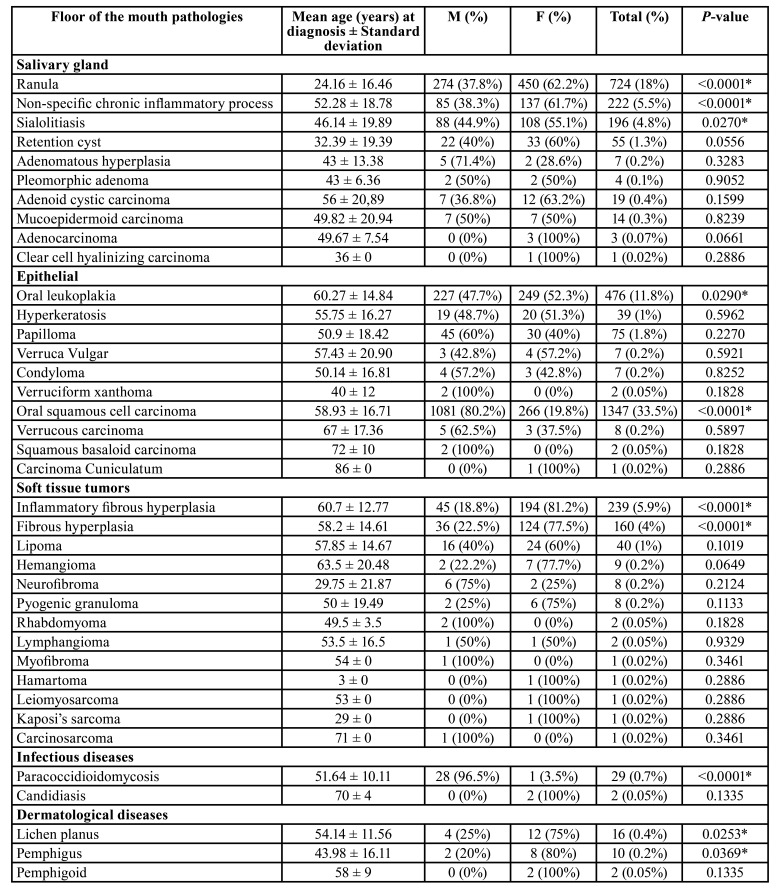
Distribution of lesions according to gender.

**Table 1 cont.**
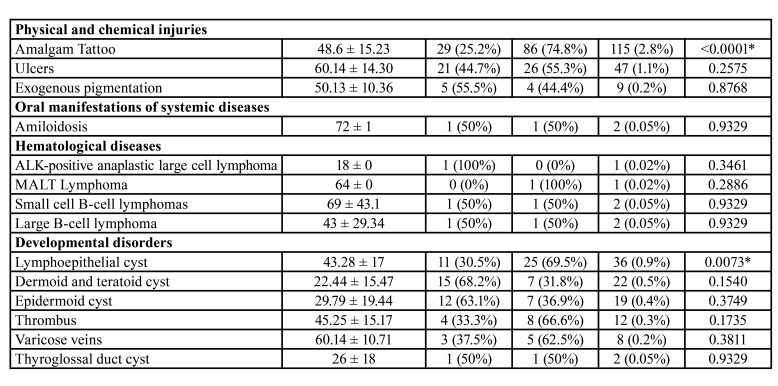
Distribution of lesions according to gender.

**Table 2:**
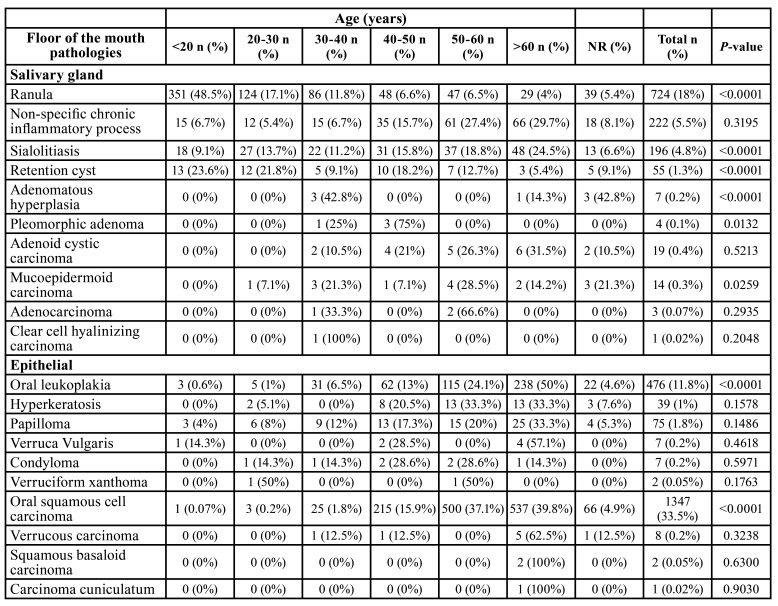
Distribution of lesions according to age.

**Table 2 cont.**
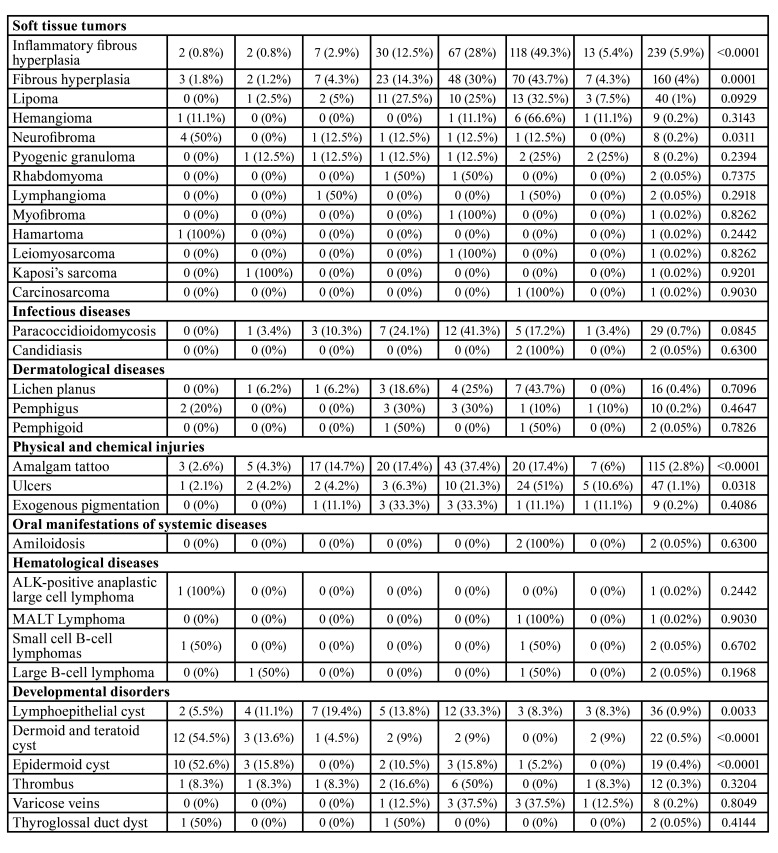
Distribution of lesions according to age.

Lesions of epithelial origin were more frequent (1,964 cases; 48.8%), followed by salivary glands (1,243 cases; 30.9%) and soft tissue (473 cases; 11.7%). The most common histological subtypes were oral squamous cell carcinoma (1,347 cases; 33.5%), ranula (724 cases; 18%), oral leukoplakia (476 cases; 11.8%) and inflammatory fibrous hyperplasia (239 cases; 5.9%). Males (2,129 cases) were more affected than females (1,887 cases). The patients demonstrated a mean age of 38.7 years old (range of 8-89 year old).

**Figure 1:**
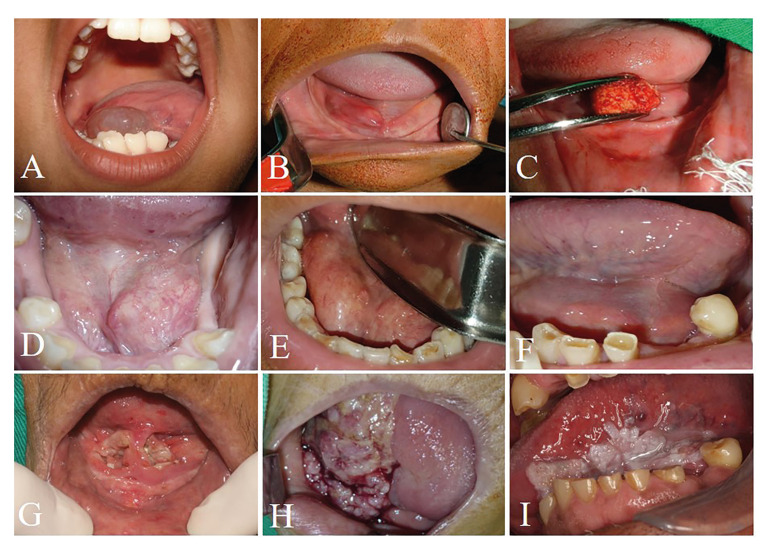
Clinical aspect of salivary gland and epithelial lesions. A) A 9-year-old male patient was referred with a large unilateral translucent mass diagnosed as ranula. B) A 64-year-old female patients was presented with a painful swelling with obstruction of submandibular duct diagnosed with sialolithiasis. C) Surgical procedure of sialolith removal. D) A 58-year-old female patient with a normal-colored asymptomatic swelling diagnosed as adenoid cystic carcinoma. E) A 44-year-old female patient was presented with a painful swelling in the floor of the mouth posteriorly diagnosed as adenoid cystic carcinoma. F) A 65-year-old female patient was referred with an asymptomatic swelling diagnosed as mucoepidermoid carcinoma. G) A 92-year-old female patient was presented with an ulcerated bilateral lesion in the floor of the mouth diagnosed as oral squamous cell carcinoma. H) A 68-year old female patient with an ulcerated lesion in the lateral border of the tongue, with invasion of the floor of the mouth. I) A 71-year-old male patient was referred with a white plaque diagnosed as oral leukoplakia.

Salivary gland lesions (SGL) represented 1,245 cases (31%) of the sample. Ranula (724 cases; 18%) (Fig. 1), non-specific chronic inflammatory process (222 cases; 5.5%) and sialolithiasis (196 cases; 4.8%) (Fig. 1) were the most common lesions. Adenoid cystic carcinoma (19 cases; 0.4%) (Fig. 1) and mucoepidermoid carcinoma (14 cases; 0.3%) (Fig. 1) were the most common malignant lesions. Patients affected by this group of lesions were mostly in age lower than 20 years old (397 cases) and female (755 cases) were more affected than male (490 cases) with a M:F ratio of 1:1.54. Statistical analysis revealed that ranula (P-value: <0.0001), non-specific chronic inflammatory process (P-value: <0.0001) and sialolithiasis (P-value: 0.0270) were statistically associated with female patients. Regarding the correlation between age and lesion, patients younger than 20 years old were statistically correlated with ranula (P-value: <0.0001) and retention cyst (P-value: <0.0001), as well as between 30 and 40 years old with adenomatous hyperplasia (P-value: <0.0001), individuals between 40 and 50 years old with pleomorphic adenoma (P-value: 0.0132), between 50-60 years old with mucoepidermoid carcinoma (P-value: 0.0259), and patients older than 60 years old were statistically correlated with sialolithiasis (P-value: <0.0001).

Epithelial lesions (EL) were presented in (1,964 cases; 48.9%). Oral squamous cell carcinoma (1,347 cases; 33.5%) (Fig. 1), oral leukoplakia (476 cases; 11.8%) (Fig. 1) and papilloma (75 cases; 1.8%) were the most frequently diagnosed lesions. Individuals older than 60 years old (826 cases) and males (1,388 cases) were more affected than females (576 cases) in a M:F ratio of 2.4:1. Females were statistically correlated with oral leukoplakia (P-value: 0.0290) and males with oral squamous cell carcinoma (P-value: <0.0001). In concern of age and lesion, it was observed that oral leukoplakia (P-value: <0.0001) and oral squamous cell carcinoma (P-value: <0.0001) were statistically related with patients older than 60 years old.

**Figure 2:**
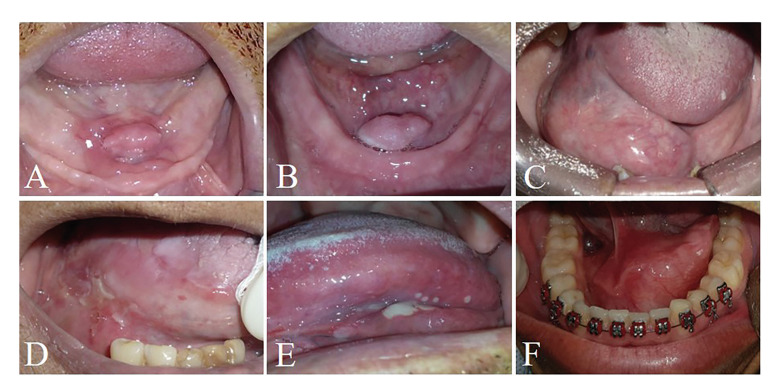
Clinical aspect of dermatological diseases, physical and chemical injuries, oral manifestation of systemic diseases and developmental disorders. A) A 55-year-old female patient was presented with a painful nodule in the floor of the mouth associated with the use of prosthesis diagnosed as inflammatory fibrous hyperplasia. B) A 81-year-old was affected by a normal-coloured swelling diagnosed as lipoma. C) A 60-year-old male patient was referred with a nodule in the floor of the mouth diagnosed as fibrous hyperplasia. D) A 55-year-old female patients was presented with an ulcerated lesion in the floor of the mouth also affecting the lateral border of the tongue which was diagnosed as pemphigus. E) A 56-year-old male patient was referred with multiple ulcers causing burning during feeding diagnosed as recurrent aphthous ulceration. F) A 19-year-old male patient was referred with an asymptomatic swelling diagnosed as epidermoid cyst.

Soft tissue tumors (STT) represented 475 cases (11.7%). Inflammatory fibrous hyperplasia (239 cases; 5.9%) (Fig. 2), fibrous hyperplasia (160 cases; 4%) (Fig. 2) and lipoma (40 cases; 1%) (Fig. 2) were the most frequent lesions of this group. Individuals older than 60 years were the most frequently affected by this group of lesions (212 cases) and females (363 cases) were more commonly detected than males (112 cases) in a M:F ratio of 1:3.2. Statistical evaluation evidenced inflammatory fibrous hyperplasia (P-value: <0.0001) and fibrous hyperplasia (P-value: <0.0001) statistically associated with female patients. In addition, individuals older than 60 years old were also statistically related with inflammatory fibrous hyperplasia (P-value: <0.0001) and fibrous hyperplasia (P-value: 0.0001), as well as patients younger than 20 years old were associated with neurofibroma development (P-value: 0.0311).

Infectious diseases (ID) were seen in 31 cases (0.7%). The two lesions observed in the current group were paracoccidioidomycosis (29 cases; 0.7%) and candidiasis (2 cases; 0.05%), and mostly affected patients with ages from 50 to 60 years old (12 cases). Males (28 cases) were affected more frequently than females (3 cases) with a M:F ratio of 1:9.3. Male patients were statistically correlated with paracoccidioidomycosis (P-value: <0.0001).

Dermatological diseases (DEDI) were observed in 28 cases (0.69%). Lichen planus (16 cases; 0.4%), pemphigus vulgaris (10 cases; 0.2%) (Fig. 2) and pemphigoid (2 cases; 0.05%) were the DEDI observed in the FOM. Patients affected by this group of lesions mainly were older than 60 years old (9 cases) and females (22 cases) were more affected than males (6 cases). Statistical analysis evidenced that females were statistically associated with lichen planus (P-value: 0.0253) and pemphigus (P-value: 0.0369).

Physical and chemical injuries (PCI) represented 171 cases of the sample (4.2%). The lesions detected on this group were amalgam tattoo (115 cases; 2.8%), ulcers (47 cases; 1.1%) (Fig. 2) and exogenous pigmentation (9 cases; 0.2%). Individuals with age ranging from 50 to 60 years old were mostly identified (56 cases) and females (116 cases) were more affected than male (55 cases), with a M:F ratio of 1:2.1. Female patients were statistically associated with amalgam tattoo (P-value: <0.0001). In addition, patients between 50 and 60 years old were statistically related with amalgam tattoo (P-value: <0.0001) and patients with age higher than 60 years old with ulcers (P-value: 0.0318).

Oral manifestation of systemic diseases represented two cases and all were patients affected by amyloidosis on the FOM with age older than 60 years old. No sex predilection was observed. No significant result was seen in this group when the statistical analysis was performed.

Hematological diseases (HD) were six cases (0.14%) and small cell B-cell lymphomas (2 cases; 0.05%) and large B-cell lymphoma (2 cases; 0.05%) were the most common histological types. Patients with age higher than 60 years old represented three cases. This group of lesions did not demonstrate any gender predominance. No significant result was evidenced in the present group in the statistical analysis.

Developmental disorders (DD) were seen in 99 cases (2.4%). Lymphoepithelial cyst (36 cases; 0.9%), dermoid and teratoid cyst (22 cases; 0.5%) and epidermoid cyst (19 cases; 0.4%) (Fig. 2). People with age lower than 20 years old and ranging from 50 to 60 years old were mostly seen, representing 26 cases each group. DD demonstrated a slight frequency in females (53 cases) than males (46 cases), with a M:F ratio of 1:1.15. Female patients were statistically correlated with lymphoepithelial cyst (P-value: 0.0073). Moreover, dermoid and teratoid cyst (P-value: <0.0001) and epidermoid cyst (P-value: <0.0001) were statistically associated with patients younger than 20 years old, as well as lymphoepithelial cyst was related with patients between 50 and 60 years old (P-value: 0.0033).

## Discussion

The findings of the current study showed that 3.49% of the analysed cases were presented in the FOM. In a systematic review of oral cancer in Arab countries, Al-Jaber and collaborators observed that FOM was affected in 50% of all sample (3). In addition, a study developed in Zimbabwe observed that 18.5% of cases were lesions located in the FOM (8). Nemes *et al*. investigated the prevalence of oral lesions in Northeastern Hungary and showed that 27.7% affected the FOM (9). These differences seem to be related to the number of FOM lesions included in each study, and the number of individuals examined. Besides differences in age, sex and habitual differences between populations, methodology and criteria of diagnosis could influence the results. Thus, this study aimed to explore the clinical and histopathological observation of the lesions affecting the FOM in Latin America, as well as investigate whether the age and gender of the patient raise the diagnostic hypothesis of each lesion.

In the current study, SGL represented 31% of all lesions in the FOM. In a Chinese epidemiological study, it was observed that SGL in the FOM represented 1.5% of all samples (10). In the current study, ranula, non-specific chronic inflammatory process and sialolithiasis were most common benign lesions. Previous studies demonstrated that pleomorphic adenoma was the most common lesion in the FOM, which is not consistent with the present research (10,11). According De Oliveira *et al*. SGL lesions in the FOM tend to be presented as malignancies (11). In our study, malignant lesions represented 2.9% of all SGL and adenoid cystic carcinoma was the most common histological type. Clinically, SGL tended to affect female patients in age lower than 20 years old. Li *et al*. observed that 1.5% of lesions affected the FOM and male patients were more frequently seen, which is in contrast of the present study (10).

EL were observed in 48.9% and oral squamous cell carcinoma (1,347 cases), oral leukoplakia (476 cases) and papilloma (75 cases) were most commonly seen. A Brazilian study observed that EL in the FOM comprised just 9.5% of all sample (12). Under clinical evaluation, males older than 60 years old were more affected by this group of lesions (13). In addition, a previous study of our group showed that oral squamous cell carcinoma of the FOM affected males older than 60 years old (14). It is important to emphasize that leukoplakia had higher mean ages than the average age of the patients affected by oral squamous cell carcinoma, suggesting that the majority of oral squamous cell carcinoma was not originated from pre-existing leukoplakias.

STT were observed in 11.7% of cases and inflammatory fibrous hyperplasia (239 cases), fibrous hyperplasia (160 cases) and lipoma (40 cases) were the most common lesions seen on this study. In Venezuela, a study exploring oral lesions in elderly people showed that STT represented 18% of all analysed sample (15). Clinical aspects evidenced that females older than 60 years old were more affected, consistent with previous epidemiological studies in Brazil, Chile and Mexico (16,17).

ID were seen in 0.7% of the analysed samples and paracoccidioidomycosis (29 cases) was the most common entity found this group of lesions. Two of the centres included this group are located in an endemic region for paracoccidioidomycosis. Dutra and collaborators observed that FOM was not a common location for ID, representing just 2% of all cases of paracoccidioidomycosis of their sample (18). Under clinical evaluation, ID affected males with age ranging from 50 to 60 years old, consistent with previous literature (19).

DEDI were shown in 0.6% of the present samples and presented lichen planus (16 cases), pemphigus (10 cases) and pemphigoid (2 cases) as the most common lesion in the FOM. Sultan *et al*. demonstrated that pemphigus is the most common dermatological lesions with oral manifestation in USA (20). Clinically, lesions tended to be presented in females older than 60 years old, as well as previous studies (21). In addition, this group of lesions is not commonly observed in the FOM (22).

PCI corresponded to 4.2% of our cases and the mostly observed lesions were amalgam tattoo (115 cases), ulcers (47 cases) and exogenous pigmentation (9 cases). A Brazilian study observed that amalgam tattoo was also the most common lesion and that FOM was not a frequent location to observe PCI (23). In the current study, females were more affected at an age ranging from 50 to 60 years old (23,24).

Oral manifestation of systemic diseases were also reported in our study and we had two cases of amyloidosis affecting the floor of the mouth. A Latin America study showed that FOM is affected in 14.3% of all cases. Under clinical evaluation, this group of lesions commonly affects patients older than 60 years old and demonstrated no gender predominance. Previous literature showed that males with a peak age of 50 years old are more described (25).

Regarding HD, they were uncommon lesions in the FOM, representing just 0.14% of all lesions. Deng *et al*. showed that lymphomas are rare on the FOM (26). Clinically, our study demonstrated that these lesions affect patients on age lower than 20 years old and higher than 60 years old, consistent with previous literature (27).

DD were observed in 2.4% of all cases seen in the current study and lymphoepithelial cyst (36 cases), dermoid and teratoid cyst (22 cases) and epidermoid cyst (19 cases) were the most common lesions. Dovigi and colleagues recognized that in USA, DD corresponded to 6.5% of 51,781 cases analysed on their study (28). In the present research, the patients of this group of lesions showed a slight female frequency, and people with age younger than 20 years old and ranging from 50 to 60 years old were mostly seen. Nonaka *et al*. observed that females in the fourth decade of life were more affected by this group of lesions, consistent with our results (29).

Interestingly, the present study showed that lesions in the FOM show a tendency towards malignancy both at the epithelial and glandular levels, which highlights the need for care for patients affected by lesions in this anatomical location (30). In conclusion, this manuscript shows the trends of the oral pathologies of the FOM, and oral squamous cell carcinoma, ranula and leukoplakia were the most common histological types. In conclusion, this manuscript shows the trends of the oral pathologies of the FOM, and oral squamous cell carcinoma, ranula and leukoplakia were the most common histological types.
